# Prenatal allostatic load and preterm birth: A systematic review

**DOI:** 10.3389/fpsyg.2022.1004073

**Published:** 2022-10-04

**Authors:** Shahirose Sadrudin Premji, Gianella Santos Pana, Alexander Cuncannon, Paul E. Ronksley, Aliyah Dosani, K. Alix Hayden, Sharifa Lalani, Joseph Wangira Musana, Kiran Shaikh, Ilona S. Yim, Maternal-infant Global Health Team (MiGHT) Collaborators in Research

**Affiliations:** ^1^School of Nursing, Faculty of Health, York University, Toronto, ON, Canada; ^2^School of Nursing and Midwifery, Faculty of Health, Community and Education, Mount Royal University, Calgary, AB, Canada; ^3^Addiction and Mental Health, Alberta Health Services, Calgary, AB, Canada; ^4^Department of Community Health Sciences, University of Calgary, Calgary, AB, Canada; ^5^Libraries and Cultural Resources, University of Calgary, Calgary, AB, Canada; ^6^School of Nursing and Midwifery, Aga Khan University, Karachi, Pakistan; ^7^Department of Obstetrics and Gynecology, Aga Khan University Hospital, Nairobi, Kenya; ^8^Department of Psychological Science, University of California, Irvine, Irvine, CA, United States

**Keywords:** prenatal allostatic load, stress, pregnancy, premature birth, perinatal mental health, perinatal distress, MiGHT

## Abstract

**Objective:**

Allostatic load refers to cumulative neuroendocrine burden and has been postulated to mediate and moderate physiological and psychological stress-related responses. This may have important implications for the risk of preterm birth. This systematic review examines the evidence on the association between prenatal allostatic load and preterm birth.

**Data sources:**

A comprehensive search of seven electronic databases was conducted from inception to August 23, 2022 to identify all English-language observational and mixed methods studies examining allostatic load and preterm birth with no year or geographic restrictions.

**Study eligibility criteria:**

Studies were included if they measured allostatic load, evaluated as the cumulative effect of any combination of more than one allostatic load biomarker, during pregnancy. Studies must have observed preterm birth, defined as < 37 weeks' gestational age, as a primary or secondary outcome of interest.

**Study appraisal and synthesis methods:**

The Quality In Prognosis Studies tool was used to evaluate risk of bias within included studies. A narrative synthesis was conducted to explore potential associations between allostatic load and preterm birth, and sources of heterogeneity.

**Results:**

Three prospective cohort studies were identified and revealed mixed evidence for an association between allostatic load and preterm birth. One study reported a statistically significant association while the other two studies reported little to no evidence for an association. Heterogeneity in when and how allostatic load was measured, limitations in study design and cohort socio-demographics may have contributed to the mixed evidence.

**Conclusions:**

This review provides insight into key individual-, community-, and study-level characteristics that may influence the association between allostatic load and preterm birth. Knowledge gaps are identified as foci for future research, including heterogeneity in allostatic load biomarkers and allostatic load index algorithms as well as pregnancy-specific considerations for allostatic load measurement. Further investigation of the allostatic load framework in the context of perinatal mental health is needed to advance understandings of maternal, infant, and child health.

**Systematic review registration:**

https://www.crd.york.ac.uk/prospero/display_record.php?ID=CRD42020208990, PROSPERO, identifier: CRD42020208990.

## Introduction

Preterm infants (babies born prior to 37 weeks' gestation), often encounter complications of prematurity (e.g., hyperbilirubinemia, hypothermia, necrotizing enterocolitis, respiratory distress, and sepsis) that are associated with higher preterm-related morbidity and mortality rates when compared to infants born at term (Purisch and Gyamfi-Bannerman, 2017; World Health Organization, 2018). Preterm birth can also have consequences for morbidity across the lifespan (e.g., psychiatric morbidity, academic problems, and social adversity) (D'Onofrio et al., 2013), which can strain families, communities, and health care systems. Identifying ways to predict and prevent preterm birth are required to reduce the aforementioned burden and contribute to resilient families, communities, and healthcare systems to ensure that every child survives and thrives to attain their full potential (UNICEF, 2019).

Although the etiology for preterm birth is multifactorial, observational research (Staneva et al., 2015) suggests that maternal stress and stress-related responses (e.g., depression and anxiety) during pregnancy increase the overall risk of preterm birth (Simmons et al., 2010). As a result, there has been growing interest in studying stress-related pathways of preterm birth. Stress and stress-related responses initiate varying degrees of multi-system physiologic responses by the body to overcome challenges or events, both predictable and unpredictable, encountered over the course of life within varied structural, social, cultural, and environmental contexts (McEwen and Wingfield, 2003; Offidani et al., 2013). McEwen and Stellar (1993) described allostatic load (AL) as the cumulative physiological and psychological “wear-and-tear” on body systems (e.g., neuroendocrine, immune, metabolic, and cardiovascular systems) resulting from repeated adaptations to challenges or events over time (Juster et al., 2010). The effect of stress and stress-related responses on preterm birth vary by geographic location (e.g., resource-poor vs. resource-rich countries) (Grote et al., 2010), socioeconomic status (Grote et al., 2010), types of maternal stress and stress-related responses, and periods of gestation (Sandman et al., 2012; Staneva et al., 2015). AL provides a plausible explanation for these differences (Premji and MiGHT, 2014; Premji et al., 2015; Riggan et al., 2021).

A recent systematic review that synthesized all available evidence through December 2019 on AL and impact on health, established an increased susceptibility to poor health from higher AL among clinical or non-clinical adult populations (Guidi et al., 2021). For example, an association was found between AL and decline in cognitive and physical functioning among older adults (Guidi et al., 2021). Although reference was made to studies examining AL in perinatal individuals (Hux et al., 2014; Accortt et al., 2017; Shalowitz et al., 2019), these studies were not critically synthesized to determine the extent to which the risk for preterm birth and other adverse pregnancy outcomes increases when AL exceeds the ability of the individual to cope (Olson et al., 2015). Given this knowledge gap, we conducted a systematic review to summarize available evidence on the association between prenatal AL and preterm birth.

## Methods

This systematic review presents results in accordance with the PRISMA 2020 guidelines (Page et al., 2021) and follows a pre-specified study protocol (PROSPERO: CRD42020208990). The PRISMA 2020 Main Checklist is shown in Supplementary material 1.

### Search strategy

The search strategy was developed by a research librarian (KAH) with experience in conducting systematic reviews with input from a clinical expert (SSP), and integrated feedback from experts in the field. Supplementary material 2 details the complete search strategies. The search strategy focused on two main search concepts: AL, both as a measure of stress as well as the effect of an accumulation of stress biomarkers, and preterm birth, defined as birth < 37 weeks' gestation and which may be operationalized as a categorical (i.e., preterm birth at < 37 weeks' gestation or term birth at ≥37 weeks' gestation) or continuous (i.e., birth from 24 to 36 6/7 weeks' gestation) variable in studies. The search was limited to English-language studies and excluded animal studies.

Databases that were searched included MEDLINE^®^ and Epub Ahead of Print, In-Process and Other Non-Indexed Citations and Daily (OVID), Embase (OVID), Cochrane Central Register of Controlled Trials (OVID), APA PsycINFO (OVID), CINAHL Plus with Full Text (Ebsco), Scopus (Elsevier), and Web of Science Core Collection. Databases were searched from inception to August 23, 2022 with no restrictions by year or geographic location. Additional papers were identified through backward searches (reference list of included studies) and forward searches (snowballing) of studies cited in the included papers.

### Selection process

Records were uploaded and screened within Covidence^®^, a web-based software for systematic reviews. Inclusion criteria for title and abstract screening were studies that focused on AL evaluated as the cumulative effects of any combination of AL biomarkers (i.e., more than one regulatory system). AL was characterized as measuring physiological dysregulation across multiple systems (e.g., cardiovascular, immune, metabolism, neuroendocrine) (McEwen and Wingfield, 2003). As there is no standardized approach to calculate a summary measure of the AL biomarkers (Li et al., 2019), restrictions were not applied with regards to the statistical strategies employed in computing AL. English-language studies were included only if they measured AL in the pregnant participant and the measurements were taken during the prenatal period. Therefore, studies were excluded if they only measured preconception or postpartum AL in pregnant participants, or if AL was only measured in preterm infants. Studies were included if they observed preterm birth as an outcome of interest (primary or secondary). Prospective and retrospective cohort studies, case-control studies, cross-sectional studies, and mixed-methods studies were included. Animal studies, editorials, letters, reviews, books, book chapters, and commentaries were excluded. Conference abstracts were included only if the authors published their work and there was extractable data that could be included in this review.

Crowdsourcing from the Maternal-infant Global Health Research Team (MiGHT) resulted in eight reviewers (SSP, GSP, AC, AD, SL, JWM, KS, ISY) involved at different points in the review process. Prior to screening within Covidence, the inclusion and exclusion criteria were evaluated by screening groups of 50 randomly selected title and abstracts on Microsoft Excel^®^ (Microsoft, 2021) until a percent agreement of 80% was reached. After screening the first 50 titles and abstracts, the percent agreement between two reviewers (SSP, GSP) was 98%. Within Covidence, each title and abstract were screened by two of six independent reviewers (GSP, AD, SL, JWM, KS, ISY), with one reviewer screening them all (GSP) and the rest screening against them (AD, SL, JWM, KS, ISY). Any conflicts at this level were resolved by a third team member (SSP). The percent agreement at title and abstract screening was 88%. The titles and abstracts that moved onto the full-text screening were retrieved and read in full by two of six independent reviewers (SSP, AD, SL, JWM, KS, ISY), with one reviewer (SSP) screening them all and the rest screening against them (AD, SL, JWM, KS, ISY). Any conflicts at this level were resolved by a smaller group of reviewers (SSP, GSP). The percent agreement at full-text screening was 83%.

### Data extraction

The main exposure variable was prenatal AL. We extracted all AL biomarkers and algorithms reported in included studies. The primary outcome of interest was preterm birth, defined as birth < 37 weeks' gestation with gestational age determined using ultrasound measurement. We extracted both categorical and continuous outcome data.

Two reviewers (AC, GSP) independently reviewed all included studies and extracted data into a standardized Microsoft Excel^®^ (Microsoft, 2021) spreadsheet. Data extracted included study characteristics and design, inclusion and exclusion criteria, recruitment, sample size, sociodemographic characteristics, and AL measurement including timing, AL biomarkers, and AL index algorithm. Conflicts were resolved through discussion to reach consensus. The percent agreement at data extraction was 98%. Authors were contacted by email if items were unclear or missing; we clarified the sample size of one study (Wallace and Harville, 2013) with its authors. Studies were examined for overlapping data and no such instances were found.

### Study risk of bias assessment

We used the Quality In Prognosis Studies (QUIPS) tool (Hayden et al., 2013) to evaluate the included studies. Two reviewers (AC, SSP) independently assessed risk of bias, ranging from low to high risk, across six QUIPS domains: study population, study attrition, prognostic factor measurement, outcome measurement, study confounding, and statistical analysis and reporting. For prognostic factor (i.e., AL) measurement, we also considered studies' abstraction of secure clinical records or direct measurement as well as the inclusion of biomarkers and alignment of AL index algorithm with previous research on AL (Juster et al., 2010). For outcome (i.e., preterm birth) measurement, we also considered studies' abstraction of secure clinical records and integration of ultrasound dating in the determination of gestational age. Disagreements were resolved through discussion to reach consensus.

### Synthesis

Although a meta-analysis was originally planned, the included studies differed substantially with respect to study design, biomarker measurement, and algorithms used to generate a composite measure of AL. Consequently, a narrative synthesis and summary of important descriptive information from included studies are presented. Data used to calculate summary measures, unadjusted and adjusted effect estimates (e.g., mean differences), and other measures of association (e.g., regression coefficients) were extracted from included studies and are contextualized in the tables and narrative synthesis. For studies that used multiple linear regression models, only data from final models are presented.

## Results

### Study selection

**Figure 1:**
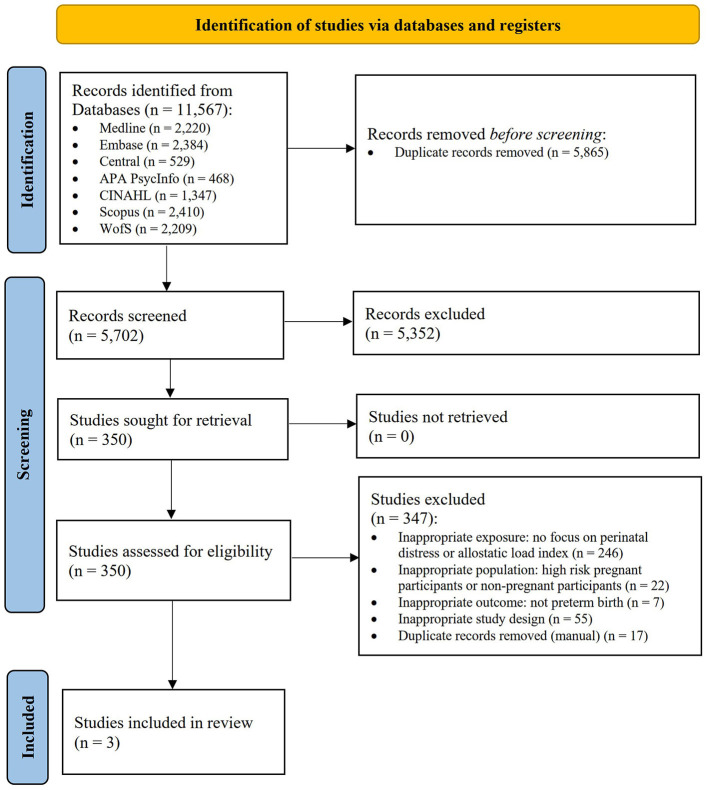
PRISMA flow diagram (Page et al., 2021).

The comprehensive search strategy identified 11,567 citations, of which 5,865 records were duplicates (shown in Figure 1). Five thousand seven hundred two title and abstracts were screened, and 350 full-text reports were retrieved and assessed for eligibility. Of these, three studies were included in the review. The most common reasons for exclusion were lack of focus on perinatal distress or the AL index (*n* = 246), inappropriate study population (*n* = 22), and inappropriate study design (*n* = 55). Several studies (Coussons-Read et al., 2012; Kramer et al., 2013; Gillespie et al., 2021; Huang et al., 2021; Keenan-Devlin et al., 2021, 2022) initially appeared promising; however, these studies did not meet all inclusion criteria and lacked connection to the AL framework as well as pathways of perinatal stress and distress, and were therefore excluded.

### Study characteristics

Of the three included studies, two were prospective cohorts (Wallace and Harville, 2013; McKee et al., 2017) and one was a doctoral thesis with secondary analysis of a prospective cohort (Sayre, 2016) (Table 1). All three studies examined gestational age as an outcome of interest in addition to other infant birth outcomes. The three studies examined cohorts ranging from 42 to 156 pregnant participants who received prenatal clinic care in the United States. Biomarkers were measured at prenatal care visits between 28 and 40 weeks' gestation (McKee et al., 2017), during each trimester (Sayre, 2016), and between 26 and 28 weeks' gestation (Wallace and Harville, 2013), respectively.

**Table 1:** Characteristics of included studies.

**Study**	**Study design**	**Setting and sample**	**Allostatic load biomarkers**	**Biomarker measurement**	**Outcomes of interest**
McKee et al. (2017)	Prospective cohort	New York, USA *n* = 111 Participants drawn from pregnant people enrolled in e-Moms of Rochester randomized clinical trial and pregnant people from one of two obstetric clinics in Rochester	8 biomarkers: • BMI • CRP • 1-hour OGTT • Cholesterol • IL-6 • SBP • DBP • Urinary albumin	At a prenatal care visit for collection of dried blood spots: • 28–40 weeks' gestation	• Gestational age (continuous) • Infants' birth weight
Sayre (2016)	Secondary analysis of prospective cohort	Kentucky and Virginia, USA *n* = 156^a^ Original data obtained at prenatal clinics affiliated with the University of Kentucky and University of Virginia	7 biomarkers: • BMI • CRP IL-1β • IL-6 • IL-10 • SBP • DBP	At prenatal care visits during each trimester: • 5–13 weeks' gestation • 14–26 weeks' gestation • 27–36 weeks' gestation	• Gestational age (continuous and categorical with PTB defined as < 37 weeks' gestation)
Wallace and Harville (2013)	Prospective cohort	Louisiana, USA *n* = 42 Prenatal clinics at Tulane-Lakeside Hospital	5 biomarkers: • Cortisol • DHEA-S • Cholesterol • HbA1c • SBP	At a prenatal care visit and scheduled glucose tolerance test: • 26–28 weeks' gestation	• Gestational age (continuous) • Infants' birth weight, birth length, and head circumference

BMI, body mass index; BP, blood pressure; CRP, C-reactive protein; DHEA-S, dehydroepiandrosterone sulfate; HbA1c, glycosylated hemoglobin; IL, interleukin; OGTT, oral glucose tolerance test; PTB, preterm birth; USA, United States of America.

^*a*^One hundred fifty-six participants had available blood pressure data in any trimester. After accounting for missing data, the multiple linear and logistic regression models for the length of gestation on AL index and preterm birth on AL index, respectively, during each trimester range from n = 74 to n = 90 participants.

The range of AL biomarkers measured varied considerably (Table 2). The studies included 8, 7, and 5 biomarkers spanning anthropometric, neuroendocrine, immune, metabolic, cardiovascular, and renal body systems. All studies included systolic blood pressure as a biomarker. Wallace and Harville's (2013) study was the only one to include neuroendocrine biomarkers (i.e., cortisol and dehydroepiandrosterone sulfate). McKee et al. (2017) and Sayre (2016) included immune biomarkers: interleukin (IL)-6 and C-reactive protein (CRP), and IL-1β, IL-6, IL-10, and CRP, respectively. McKee et al. (2017) and Wallace and Harville (2013) included metabolic biomarkers: 1-hour oral glucose tolerance test (1-hour OGTT) and cholesterol, and glycosylated hemoglobin and 1-hour OGTT, respectively.

**Table 2:** Allostatic load biomarkers measured in included studies.

	**McKee et al. (2017)**	**Sayre (2016)**	**Wallace and Harville (2013)**
Anthropometric			
BMI	•	•	
Neuroendocrine			
Cortisol			•
DHEA-S			•
Immune			
IL-1β		•	
IL-6	•	•	
IL-10		•	
CRP	•	•	
Metabolic			
HbA1c			•
1-hour OGTT	•		•
Cholesterol	•		
Cardiovascular			
Systolic BP	•	•	•
Diastolic BP	•	•	
Renal			
Urinary albumin	•		

BMI, body mass index; BP, blood pressure; CRP, C-reactive protein; DHEA-S, dehydroepiandrosterone sulfate; HbA1c, glycosylated hemoglobin; IL, interleukin; OGTT, oral glucose tolerance test.

### Results of individual studies and syntheses

The analyses and findings of the included studies on the association between AL index and gestational age or preterm birth are shown in Table 3. McKee et al. (2017) framed AL as reflecting cumulative physiologic dysfunction (CPD) and used a count-based method in which each biomarker within a high-risk quartile was counted as one point in a composite CPD score, akin to an AL index. Similarly, Sayre (2016) used a count-based method with high-risk tertiles. Wallace and Harville (2013) generated an AL index from a summation of five z-scores in which each biomarker was standardized based on the total sample distribution, or for cortisol, sample mean at the time of venipuncture.

**Table 3:** Allostatic load scoring, findings, and analyses of included studies.

**Study**	**AL index scoring: Algorithm Mean AL index**	**Outcomes:** **Mean length of gestation** **PTB rate**	**Adjustments**	**Association between AL index and length of gestation or PTB**
McKee et al. (2017)	Count-based method based on high-risk quartiles Mean = 2.09 SD = 1.42	39.64 weeks (SD = 1.08 weeks) 1.8%	• Race • Income • Smoking • Education • Relationship status	CPD score was not a statistically significant predictor of length of gestation. CPD score in adjusted LR model: β = −0.06, p = 0.44
Sayre (2016)	Count-based method based on high-risk tertiles First-trimester mean = 2.05 Second-trimester mean = 2.18 Third-trimester mean = 2.13	38.88 weeks (range = 25.29–41.57 weeks) 10%	• Gravidity	Third-trimester AL index was a statistically significant predictor of length of gestation among term births only. Third-trimester AL index in adjusted LR model: B = −1.38, β = −0.25, *p* < 0.05 AL index was not a statistically significant predictor of preterm birth in any trimester in logistic regression models.
Wallace and Harville (2013)	Z-score method Mean = 0.2 SD = 1.7	38.9 weeks (SD = 1.5 weeks) PTB rate not reported	• BMI • Tobacco use	AL index was a statistically significant predictor of length of gestation. AL index in adjusted LR model: β = −0.18; 95% CI = [−0.35, 0.00]; *p* = 0.05

AL, allostatic load; CI, confidence interval; CPD, cumulative physiologic dysfunction; LR, linear regression; PTB, preterm birth; SD, standard deviation.

The average length of gestation ranged from 38.88 to 39.64 weeks among the cohorts. McKee et al. (2017) and Sayre (2016) reported preterm birth rates of 1.8 and 10%, respectively, for their cohorts. In the cohort studied by Wallace and Harville (2013), the rate of preterm birth was not reported. Covariate adjustment within final models varied among the studies, with no overlap except for tobacco use in both the McKee et al. (2017) and Wallace and Harville (2013) cohorts.

All three included studies used multiple linear regression to examine the association between AL index and length of gestation. In the McKee et al. (2017) cohort, cumulative physiologic dysfunction score was not associated with length of gestation (β = −0.06, *p* = 0.44). In Sayre's (2016) cohort, third-trimester AL index predicted a small amount of variance in the length of gestation among pregnant participants with term births only (*B* = −1.38, β = −0.25, *p* < 0.05), although first, second-, and third-trimester AL indexes were not statistically significant predictors of preterm birth (i.e., birth < 37 weeks' gestation) in logistic regression models. In the Wallace and Harville (2013) cohort, AL index was a statistically significant predictor of length of gestation in both unadjusted and adjusted [β = −0.18; 95% CI = (−0.35, 0.00); *p* = 0.05] models.

### Risk of bias in studies

A risk of bias assessment was completed using the QUIPS tool (Hayden et al., 2013) as shown in Supplementary material 3. We identified concerns about the impact of sample and attrition bias on representativeness in all studies. Limited information was provided in some studies regarding recruitment processes (Wallace and Harville, 2013; Sayre, 2016), cohort sociodemographic characteristics (Wallace and Harville, 2013), and attrition (Wallace and Harville, 2013; Sayre, 2016). Studies generally had restrictive sampling frames (e.g., select number of recruitment sites) and inclusion criteria (e.g., planned delivery at specific health care facilities) (Wallace and Harville, 2013; McKee et al., 2017). Some exclusion criteria were particularly restrictive (e.g., blood pressure pharmacotherapy or chronic disease diagnosis) or vague [e.g., “indication of drug abuse” (Sayre, 2016)] as shown in Supplementary material 4. These factors may have contributed to two of the cohorts (Sayre, 2016; McKee et al., 2017) being predominantly made up of participants who were white and had postsecondary levels of education.

Two studies (Sayre, 2016; McKee et al., 2017) were identified as having moderate-to-high risk of bias due to measurement. For prognostic factor (i.e., AL) measurement, the extent of missing biomarker data in some of the studies raised concerns. Specifically, biomarkers were measured inconsistently at prenatal care visits during each trimester in the cohort studied by Sayre (2016) and it was unclear how some missing data were managed. In this study, the extent of missing data required omitting cases and modifying analyses (e.g., limiting the multiple linear regression model to the association between third-trimester AL index and gestational age among full-term births). In addition, the late timing of AL biomarker measurement in two studies (Sayre, 2016; McKee et al., 2017) should be noted. McKee et al. (2017) measured biomarkers from 28 to 40 weeks' gestation but reported that adjustment for gestational age in a separate analysis did not change results.

Studies were generally identified as having moderate risk of bias due to confounding and statistical analysis and reporting. Studies varied in the identification and control of important potential confounders [e.g., race, socioeconomic status, education, gravidity, and tobacco use (Staneva et al., 2015)]. In final models, McKee et al. (2017) and Wallace and Harville (2013) adjusted for smoking, Sayre (2016) adjusted for gravidity, and McKee et al. (2017) adjusted for race, income, tobacco use, and education.

## Discussion

### Principal findings

To our knowledge, this is the first systematic review to examine the association between prenatal AL and preterm birth. The three included prospective cohort studies revealed mixed evidence for an association between AL and preterm birth. There was a statistically significant association between increased AL and decreased length of gestation in the cohort studied by Wallace and Harville (2013), but there was limited to no support for an association in the cohorts studied by Sayre (2016) and McKee et al. (2017). There are likely numerous contributing factors to the mixed evidence synthesized in this review, including variability in when and how AL was measured, limitations in study design, and lack of diversity in the sociodemographic profiles of the study cohorts. Further research is needed that harmonizes AL measurement and AL index scoring and examines longer-term associations between exposure to chronic stress and birth outcomes.

### Comparison with existing literature

The findings of this review reveal some of the ways in which AL may contribute to preterm birth. For example, discussion about optimal timing of AL biomarker measurement and operationalization into a composite AL index is ongoing in the literature (Fava et al., 2010; Juster et al., 2010; Wallace and Harville, 2013; Sayre, 2016; Johnson et al., 2017; McKee et al., 2017; D'Amico et al., 2020). Heterogeneity in both AL biomarkers and AL index algorithms have been previously identified as a consequential source of variation in the evidence on AL and its impact on health outcomes (Johnson et al., 2017; D'Amico et al., 2020; Guidi et al., 2021), and such heterogeneity is evident in this review. There was considerable variation in AL biomarkers measured among the included studies. Measurement of both primary mediators (i.e., neuroendocrine and immune systems) and secondary mediators (i.e., cardiovascular and metabolic systems) is crucial to evaluate AL because of non-linear, multi-system, and independent contributions to the stress response (Juster et al., 2010; Hux et al., 2014). In addition, the AL framework is generally centered around hypothalamic-pituitary-adrenal (HPA) axis functioning (Juster et al., 2010; D'Amico et al., 2020; Guidi et al., 2021). Only the Wallace and Harville (2013) cohort included biomarkers (i.e., cortisol and dehydroepiandrosterone sulfate) of neuroendocrine and HPA axis activity. AL index algorithms also differed among the studies. Specifically, Wallace and Harville (2013) used a z-score method in which cortisol measurements were standardized relative to the sample mean at collection time and other biomarker were standardized based on sample distribution, enabling varying weights. The other studies used count-based methods with high-risk quartiles or tertiles. Recent research has more closely examined the optimal process for calculating an AL index. Liu et al. (2021) have proposed that algorithms incorporating item response theory may offer more precision than conventional count-based algorithms by enabling varying weights for each biomarker in a composite score. Future research should continue to explore optimal AL scoring and endeavor for greater consistency in AL biomarker measurement.

There was also variation in the timing of AL biomarker measurement among the included studies. Two studies included third-trimester measurements. In contrast, in the study by Wallace and Harville (2013), where a statistically significant association between AL and length of gestation was observed, AL biomarkers were measured only in the second trimester from 26 to 28 weeks' gestation. Prior work suggests that after this time point, the dampening of biological and psychological adaptations to stress protect both the pregnant individual and fetus from adverse health implications (Glynn et al., 2008; Premji et al., 2015). Therefore, the timing of AL biomarker measurement during pregnancy shapes how variance in AL may be explained in relation to chronic stress (Li et al., 2020). Given the superimposition of pregnancy-specific physiological and psychological alterations during the perinatal period, the scope and validity of the AL framework in these contexts are increasingly being examined (Morrison et al., 2013; Li et al., 2019, 2020). Li et al. (2019, 2020) have suggested that while AL measurements during pregnancy indeed reflect “true” physiological function and chronic stress exposure, gestational age and gestation-specific risk quartiles should be integrated into AL index algorithms. Doan (2021) has further suggested examining rates of change in AL during pregnancy to capture dysregulation more comprehensively than single timepoint measurements. Future research should continue to explore optimal measurement of pregnancy AL.

Components of and limitations in study design may have also contributed to the mixed evidence. In particular, the prospective cohort design and short-term data collection of the included studies warrant discussion. Although the studies were prospective cohorts, data were generally collected and analyzed at just two timepoints: once during pregnancy and once at delivery. Short-term longitudinal studies likely cannot comprehensively examine moderating and mediating effects as well as intermediate outcomes along pathways of chronic stress, AL, and allostatic overload. Evidently, longitudinal studies that follow people for substantially longer time frames are needed to comprehensively capture the burden and effects of cumulative life stress throughout the life course (D'Amico et al., 2020).

The sociodemographic profiles of the three included studies warrant discussion. Two of three cohorts were predominantly comprised of pregnant participants who were white, had post-secondary education, and received prenatal care in urban areas of the United States. Notably, higher AL has been observed in individuals, communities, and populations faced with socio-structural inequities and exclusion including racism and poverty (Geronimus et al., 2006; Shalowitz et al., 2019). Strikingly disparate rates of adverse birth and health outcomes are recognized to be the result of environmental factors given the social construction of race (Shalowitz et al., 2019; Leimert and Olson, 2020). Although the included studies touched on some of these disparities, the lack of diversity in the sociodemographic profiles of the study cohorts underscores a need for future research with participation that better reflects community and population diversity.

### Limitations

Limitations are noted across the three included studies. The findings of each study should be interpreted with consideration of the prospective cohort design, relatively small sample sizes, and the risks of selection, attrition, and measurement bias that we have discussed. This systematic review also has several limitations. Our search was limited to English-language literature, so there is a possibility that we may have missed studies. Furthermore, after screening, just three studies were included in the review. This may be explained by the relatively small but growing research field that encompasses both AL and perinatal health. In addition, multi-system interactions in the stress response are recognized in the body of work on AL (Premji and MiGHT, 2014; Olson et al., 2015; Premji et al., 2015). While this review focused on the association between AL and preterm birth specifically, we recognize a continuum of interrelated disease processes (e.g., pre-eclampsia, eclampsia, gestational diabetes, and hypertension) associated with cumulative life stress that contribute to a common pathway of adverse maternal-child health outcomes (e.g., intrauterine growth restriction, low birth weight, preterm birth, stillbirth, miscarriage, and infant death).

### Conclusions and implications

This systematic review provides an important synthesis of current literature on the association between AL and preterm birth. Although the included studies revealed mixed evidence for an association, which may have resulted from heterogeneity in AL measurement, limitations in study design, and cohort socio-demographics, this review provides insight into key individual-, community-, and study-level characteristics that may influence the association. This review also provides directions for further investigation of the AL framework in the context of perinatal mental health. We have highlighted knowledge gaps that may provide direction for future research, including a need for greater consistency in AL biomarkers and AL index algorithms and considerations when measuring AL during pregnancy.

## Data availability statement

The original contributions presented in the study are included in the article/Supplementary material, further inquiries can be directed to the corresponding authors.

## Author contributions

SSP, GSP, AC, PER, KAH, and AD conceptualized the project. KAH developed the search strategy with input from SSP, and KAH conducted the searches. SSP, GSP, AD, SL, JWM, KS, and ISY screened records. GSP and AC extracted and synthesized the data with supervision from SSP and PER. SSP, GSP, and AC drafted the paper. All authors revised the paper.

## Funding

This work was supported by (1) Canadian Institutes of Health Research (CIHR) project grant (FRN 153021; application number 376731): SSP is the nominated principal investigator, KS and ISY are principal applicants, and AD, SL, and JWM are co-applicants; and (2) Startup Grant, Faculty of Health, York University. These funding sources supported the Research Assistants who worked with the team on this systematic review. These funding sources were not involved in the study design, data collection and analysis, writing of the report, or decisions to submit the article.

## Conflict of interest

Authors GSP and AC were Research Assistants who were paid through the CIHR Project Grant and Startup Grant, Faculty of Health, York University. The remaining authors declare that the research was conducted in the absence of any commercial or financial relationships that could be construed as a potential conflict of interest.

## Publisher's note

All claims expressed in this article are solely those of the authors and do not necessarily represent those of their affiliated organizations, or those of the publisher, the editors and the reviewers. Any product that may be evaluated in this article, or claim that may be made by its manufacturer, is not guaranteed or endorsed by the publisher.
