# Supplementary material for: Prenatal allostatic load and preterm birth: A systematic review
Source: Front Psychol. 2022 Oct 4;13:1004073. doi: 10.3389/fpsyg.2022.1004073 (PMC9577361; doi:10.3389/fpsyg.2022.1004073)
Supplement: Supplementary file 2 [file Data_Sheet_2.DOCX]

Supplementary Material S2: Search strategies

Database(s): **Ovid MEDLINE(R) and Epub Ahead of Print, In-Process, In-Data-Review & Other Non-Indexed Citations and Daily**1946 to August 22, 2022

Search Strategy:

| **#** | **Searches** | **Results** |
| --- | --- | --- |
| 1 | exp Infant, Premature/ or exp Infant, Extremely Premature/ | 62816 |
| 2 | exp Premature Birth/ | 19001 |
| 3 | ((preterm* or pre-term* or prematur* or pre-matur*) adj5 (birth or births or childbirth or born or delivery or delivered or deliveries or parturition or labor or labour)).tw,kf. | 69413 |
| 4 | ((early-preterm or mild-preterm or near-term) adj5 (birth or births or childbirth or born or delivery or delivered or deliveries or parturition or labor or labour)).tw,kf. | 1096 |
| 5 | or/1-4 | 119282 |
| 6 | (("24" or "25" or "26" or "27" or "28" or "29" or "30" or "31" or "32" or "33" or "34" or "35" or "36") adj (week* or wk* or ga or gestation* or postmenstrual age or post-menstrual age)).tw,kf. | 117535 |
| 7 | (24wk* or 25wk* or 26wk* or 27wk* or 28wk* or 29wk* or 30wk* or 31wk* or 32wk* or 33wk* or 34wk* or 35wk* or 36wk*).tw,kf. | 129 |
| 8 | (24week* or 25week* or 26week* or 27week* or 28week* or 29week* or 30week* or 31week* or 32week* or 33week* or 34week* or 35week* or 36week*).tw,kf. | 1261 |
| 9 | (("less than" or before or "fewer than") adj5 ("37 week*" or "37week*" or "37wk*" or "37 wk*")).tw,kf. | 1944 |
| 10 | ("36 6/7 week*" or "36 6/7 wk*" or "36 6/7week*" or "36 6/7wk*").tw,kf. | 257 |
| 11 | or/6-10 | 120219 |
| 12 | exp Parturition/ | 20627 |
| 13 | (birth or births or childbirth or born or delivery or delivered or deliveries or parturition or labor or labour).tw,kf. | 1165015 |
| 14 | (infant* or baby or babies or newborn* or neonate*).tw,kf. | 699316 |
| 15 | or/12-14 | 1659863 |
| 16 | 11 and 15 | 53473 |
| 17 | 5 or 16 | 146941 |
| 18 | exp Allostasis/ | 903 |
| 19 | *Stress, Psychological/ | 81016 |
| 20 | *Stress, Physiological/ | 45863 |
| 21 | *Biomarkers/ | 46433 |
| 22 | (allostatic adj2 (load* or overload* or mediator*)).tw,kf. | 1482 |
| 23 | allostasis.tw,kf. | 637 |
| 24 | (stress adj3 (chronic or cumulative or antenatal or ante-natal or prenatal or pre-natal or psychosocial or maternal or psychologic* or physiologic*)).tw,kf. | 52675 |
| 25 | (cumulative adj3 (physiologic* risk or biologic* risk or psychologic* risk)).tw,kf. | 42 |
| 26 | (biomarker* adj6 (maternal or pregnan* or perinatal or prenatal or pre-natal or peri-natal or pre-natal or pre-conception or preconception or stress)).tw,kf. | 12682 |
| 27 | (biological marker* adj6 (maternal or pregnan* or perinatal or prenatal or pre-natal or peri-natal or pre-natal or pre-conception or preconception or stress)).tw,kf. | 268 |
| 28 | (two-hit* or multiple hit*).tw,kf. | 2467 |
| 29 | or/18-27 | 215705 |
| 30 | 17 and 29 | 2360 |
| 31 | limit 30 to english language | 2281 |
| 32 | limit 31 to "humans only (removes records about animals)" | 2220 |

Database(s): **Embase**1974 to 2022 August 22

Search Strategy:

| **#** | **Searches** | **Results** |
| --- | --- | --- |
| 1 | exp prematurity/ or premature labor/ | 163753 |
| 2 | ((preterm* or pre-term* or prematur* or pre-matur*) adj5 (birth or births or childbirth or born or delivery or delivered or deliveries or parturition or labor or labour)).tw,kw. | 97595 |
| 3 | ((early-preterm or mild-preterm or near-term) adj5 (birth or births or childbirth or born or delivery or delivered or deliveries or parturition or labor or labour)).tw,kw. | 1578 |
| 4 | or/1-3 | 187807 |
| 5 | (("24" or "25" or "26" or "27" or "28" or "29" or "30" or "31" or "32" or "33" or "34" or "35" or "36") adj (week* or wk* or ga or gestation* or postmenstrual age or post-menstrual age)).tw,kw. | 182157 |
| 6 | (24wk* or 25wk* or 26wk* or 27wk* or 28wk* or 29wk* or 30wk* or 31wk* or 32wk* or 33wk* or 34wk* or 35wk* or 36wk*).tw,kw. | 861 |
| 7 | (24week* or 25week* or 26week* or 27week* or 28week* or 29week* or 30week* or 31week* or 32week* or 33week* or 34week* or 35week* or 36week*).tw,kw. | 2369 |
| 8 | (("less than" or before or "fewer than") adj5 ("37 week*" or "37week*" or "37wk*" or "37 wk*")).tw,kw. | 2712 |
| 9 | ("36 6/7 week*" or "36 6/7 wk*" or "36 6/7week*" or "36 6/7wk*").tw,kw. | 507 |
| 10 | or/5-9 | 186521 |
| 11 | birth/ | 20169 |
| 12 | (birth or births or childbirth or born or delivery or delivered or deliveries or parturition or labor or labour).tw,kw. | 1484524 |
| 13 | (infant* or baby or babies or newborn* or neonate*).tw,kw. | 790501 |
| 14 | or/11-13 | 2001346 |
| 15 | 10 and 14 | 81008 |
| 16 | 4 or 15 | 228581 |
| 17 | allostasis/ | 1055 |
| 18 | chronic stress/ or chronic unpredictable stress/ | 12958 |
| 19 | *mental stress/ | 35636 |
| 20 | maternal stress/ | 3703 |
| 21 | *physiological stress/ | 14610 |
| 22 | *biological marker/ | 107608 |
| 23 | (allostatic adj2 (load* or overload* or mediator*)).tw,kw. | 1717 |
| 24 | allostasis.tw,kw. | 804 |
| 25 | (stress adj3 (chronic or cumulative or antenatal or ante-natal or prenatal or pre-natal or psychosocial or maternal or psychologic* or physiologic*)).tw,kw. | 68046 |
| 26 | (cumulative adj3 (physiologic* risk or biologic* risk or psychologic* risk)).tw,kw. | 39 |
| 27 | (biomarker* adj6 (maternal or pregnan* or perinatal or prenatal or pre-natal or peri-natal or pre-natal or pre-conception or preconception or stress)).tw,kw. | 16753 |
| 28 | (biological marker* adj6 (maternal or pregnan* or perinatal or prenatal or pre-natal or peri-natal or pre-natal or pre-conception or preconception or stress)).tw,kw. | 401 |
| 29 | (two-hit* or multiple hit*).tw,kw. | 3395 |
| 30 | or/17-29 | 236099 |
| 31 | 16 and 30 | 3354 |
| 32 | limit 31 to conference abstracts | 788 |
| 33 | 31 not 32 | 2566 |
| 34 | limit 33 to english language | 2461 |
| 35 | limit 34 to "humans only (removes records about animals)" | 2384 |

**Database:** EBM Reviews - Cochrane Central Register of Controlled Trials <July 2022>

Search Strategy

| **#** | **Searches** | **Results** |
| --- | --- | --- |
| 1 | exp Infant, Premature/ or exp Infant, Extremely Premature/ | 3,992 |
| 2 | exp Premature Birth/ | 1,710 |
| 3 | ((preterm* or pre-term* or prematur* or pre-matur*) adj5 (birth or births or childbirth or born or delivery or delivered or deliveries or parturition or labor or labour)).tw,kw. | 8,957 |
| 4 | ((early-preterm or mild-preterm or near-term) adj5 (birth or births or childbirth or born or delivery or delivered or deliveries or parturition or labor or labour)).tw,kw. | 167 |
| 5 | or/1-4 | 12,602 |
| 6 | (("24" or "25" or "26" or "27" or "28" or "29" or "30" or "31" or "32" or "33" or "34" or "35" or "36") adj (week* or wk* or ga or gestation* or postmenstrual age or post-menstrual age)).tw,kw. | 50,412 |
| 7 | (24wk* or 25wk* or 26wk* or 27wk* or 28wk* or 29wk* or 30wk* or 31wk* or 32wk* or 33wk* or 34wk* or 35wk* or 36wk*).tw,kw. | 172 |
| 8 | (24week* or 25week* or 26week* or 27week* or 28week* or 29week* or 30week* or 31week* or 32week* or 33week* or 34week* or 35week* or 36week*).tw,kw. | 666 |
| 9 | (("less than" or before or "fewer than") adj5 ("37 week*" or "37week*" or "37wk*" or "37 wk*")).tw,kw. | 501 |
| 10 | ("36 6/7 week*" or "36 6/7 wk*" or "36 6/7week*" or "36 6/7wk*").tw,kw. | 63 |
| 11 | or/6-10 | 51,255 |
| 12 | exp Parturition/ | 501 |
| 13 | (birth or births or childbirth or born or delivery or delivered or deliveries or parturition or labor or labour).tw,kw. | 118,865 |
| 14 | (infant* or baby or babies or newborn* or neonate*).tw,kw. | 63,953 |
| 15 | or/12-14 | 155,347 |
| 16 | 11 and 15 | 13,866 |
| 17 | 5 or 16 | 21,489 |
| 18 | exp Allostasis/ | 8 |
| 19 | Stress, Psychological/ | 6,359 |
| 20 | Stress, Physiological/ | 1,354 |
| 21 | Biomarkers/ | 16,065 |
| 22 | (allostatic adj2 (load* or overload* or mediator*)).tw,kw. | 58 |
| 23 | allostasis.tw,kw. | 16 |
| 24 | (stress adj3 (chronic or cumulative or antenatal or ante-natal or prenatal or pre-natal or psychosocial or maternal or psychologic* or physiologic*)).tw,kw. | 6,397 |
| 25 | (cumulative adj3 (physiologic* risk or biologic* risk or psychologic* risk)).tw,kw. | 0 |
| 26 | (biomarker* adj6 (maternal or pregnan* or perinatal or prenatal or pre-natal or peri-natal or pre-natal or pre-conception or preconception or stress)).tw,kw. | 1,773 |
| 27 | (biological marker* adj6 (maternal or pregnan* or perinatal or prenatal or pre-natal or peri-natal or pre-natal or pre-conception or preconception or stress)).tw,kw. | 80 |
| 28 | (two-hit* or multiple hit*).tw,kw. | 34 |
| 29 | or/18-27 | 29,942 |
| 30 | 17 and 29 | 540 |
| 31 | limit 30 to english language | 529 |

Database(s): **APA PsycInfo**1806 to August Week 3 2022

Search Strategy:

| **#** | **Searches** | **Results** |
| --- | --- | --- |
| 1 | premature birth/ | 6228 |
| 2 | ((preterm* or pre-term* or prematur* or pre-matur*) adj5 (birth or births or childbirth or born or delivery or delivered or deliveries or parturition or labor or labour)).tw,id. | 6826 |
| 3 | ((early-preterm or mild-preterm or near-term) adj5 (birth or births or childbirth or born or delivery or delivered or deliveries or parturition or labor or labour)).tw,id. | 56 |
| 4 | or/1-3 | 9514 |
| 5 | (("24" or "25" or "26" or "27" or "28" or "29" or "30" or "31" or "32" or "33" or "34" or "35" or "36") adj (week* or wk* or ga or gestation* or postmenstrual age or post-menstrual age)).tw,id. | 9521 |
| 6 | (24wk* or 25wk* or 26wk* or 27wk* or 28wk* or 29wk* or 30wk* or 31wk* or 32wk* or 33wk* or 34wk* or 35wk* or 36wk*).tw,id. | 63 |
| 7 | (24week* or 25week* or 26week* or 27week* or 28week* or 29week* or 30week* or 31week* or 32week* or 33week* or 34week* or 35week* or 36week*).tw,id. | 97 |
| 8 | (("less than" or before or "fewer than") adj5 ("37 week*" or "37week*" or "37wk*" or "37 wk*")).tw,id. | 92 |
| 9 | ("36 6/7 week*" or "36 6/7 wk*" or "36 6/7week*" or "36 6/7wk*").tw,id. | 22 |
| 10 | or/5-9 | 9742 |
| 11 | exp Birth/ | 17512 |
| 12 | (birth or births or childbirth or born or delivery or delivered or deliveries or parturition or labor or labour).tw,id. | 223609 |
| 13 | (infant* or baby or babies or newborn* or neonate*).tw,id. | 110502 |
| 14 | or/11-13 | 308649 |
| 15 | 10 and 14 | 4034 |
| 16 | 4 or 15 | 11510 |
| 17 | chronic stress/ | 3088 |
| 18 | psychological stress/ | 9466 |
| 19 | physiological stress/ | 3405 |
| 20 | biological markers/ | 16497 |
| 21 | (allostatic adj2 (load* or overload* or mediator*)).tw,id. | 959 |
| 22 | allostasis.tw,id. | 482 |
| 23 | (stress adj3 (chronic or cumulative or antenatal or ante-natal or prenatal or pre-natal or psychosocial or maternal or psychologic* or physiologic*)).tw,id. | 28995 |
| 24 | (cumulative adj3 (physiologic* risk or biologic* risk or psychologic* risk)).tw,id. | 24 |
| 25 | (biomarker* adj6 (maternal or pregnan* or perinatal or prenatal or pre-natal or peri-natal or pre-natal or pre-conception or preconception or stress)).tw,id. | 1173 |
| 26 | (biological marker* adj6 (maternal or pregnan* or perinatal or prenatal or pre-natal or peri-natal or pre-natal or pre-conception or preconception or stress)).tw,id. | 148 |
| 27 | (two-hit* or multiple hit*).tw,id. | 212 |
| 28 | or/17-27 | 54554 |
| 29 | 16 and 28 | 495 |
| 30 | limit 29 to english language | 468 |

**CINAHL Plus with Full Text (Ebsco)**

| **#** | **Query** | **Limiters/Expanders** | **Results** |  |
| --- | --- | --- | --- | --- |
| S1 | (MH "Infant, Premature") | Search modes - Find all my search terms | 26,281 |  |
| S2 | (MH "Childbirth, Premature") OR (MH "Labor, Premature") | Search modes - Find all my search terms | 15,743 |  |
| S3 | (MH "Outcomes of Prematurity") | Search modes - Find all my search terms | 865 |  |
| S4 | TI ( ((preterm* or pre-term* or prematur* or pre-matur*) N5 (birth or births or childbirth or born or delivery or delivered or deliveries or parturition or labor or labour)) ) OR AB ( ((preterm* or pre-term* or prematur* or pre-matur*) N5 (birth or births or childbirth or born or delivery or delivered or deliveries or parturition or labor or labour)) ) | Search modes - Find all my search terms | 27,016 |  |
| S5 | TI ( ((early-preterm or mild-preterm or near-term) N5 (birth or births or childbirth or born or delivery or delivered or deliveries or parturition or labor or labour)) ) OR AB ( ((early-preterm or mild-preterm or near-term) N5 (birth or births or childbirth or born or delivery or delivered or deliveries or parturition or labor or labour)) ) | Search modes - Find all my search terms | 502 |  |
| S6 | S1 OR S2 OR S3 OR S4 OR S5 | Search modes - Find all my search terms | 51,124 |  |
| S7 | TI ( (("24" or "25" or "26" or "27" or "28" or "29" or "30" or "31" or "32" or "33" or "34" or "35" or "36") N1 (week* or wk* or ga or gestation* or “postmenstrual age” or “post-menstrual age”)) ) OR AB ( (("24" or "25" or "26" or "27" or "28" or "29" or "30" or "31" or "32" or "33" or "34" or "35" or "36") N1 (week* or wk* or ga or gestation* or “postmenstrual age” or “post-menstrual age”)) ) | Search modes - Find all my search terms | 33,630 |  |
| S8 | TI ( (24wk* or 25wk* or 26wk* or 27wk* or 28wk* or 29wk* or 30wk* or 31wk* or 32wk* or 33wk* or 34wk* or 35wk* or 36wk*) ) OR AB ( (24wk* or 25wk* or 26wk* or 27wk* or 28wk* or 29wk* or 30wk* or 31wk* or 32wk* or 33wk* or 34wk* or 35wk* or 36wk*) ) | Search modes - Find all my search terms | 66 |  |
| S9 | TI ( (24week* or 25week* or 26week* or 27week* or 28week* or 29week* or 30week* or 31week* or 32week* or 33week* or 34week* or 35week* or 36week*) ) OR AB ( (24week* or 25week* or 26week* or 27week* or 28week* or 29week* or 30week* or 31week* or 32week* or 33week* or 34week* or 35week* or 36week*) ) | Search modes - Find all my search terms | 255 |  |
| S10 | TI ( (("less than" or before or "fewer than") N5 ("37 week*" or "37week*" or "37wk*" or "37 wk*")) ) OR AB ( (("less than" or before or "fewer than") N5 ("37 week*" or "37week*" or "37wk*" or "37 wk*")) ) | Search modes - Find all my search terms | 841 |  |
| S11 | TI ( ("36 6/7 week*" or "36 6/7 wk*" or "36 6/7week*" or "36 6/7wk*") ) OR AB ( ("36 6/7 week*" or "36 6/7 wk*" or "36 6/7week*" or "36 6/7wk*") ) | Search modes - Find all my search terms | 117 |  |
| S12 | S7 OR S8 OR S9 OR S10 OR S11 | Search modes - Find all my search terms | 34,424 |  |
| S13 | (MH "Childbirth") | Search modes - Find all my search terms | 11,871 |  |
| S14 | TI ( (birth or births or childbirth or born or delivery or delivered or deliveries or parturition or labor or labour) ) OR AB ( (birth or births or childbirth or born or delivery or delivered or deliveries or parturition or labor or labour) ) | Search modes - Find all my search terms | 322,270 |  |
| S15 | TI ( (infant* or baby or babies or newborn* or neonate*) ) OR AB ( (infant* or baby or babies or newborn* or neonate*) ) | Search modes - Find all my search terms | 185,205 |  |
| S16 | S13 OR S14 OR S15 | Search modes - Find all my search terms | 445,929 |  |
| S17 | S12 AND S16 | Search modes - Find all my search terms | 18,653 |  |
| S18 | S6 OR S17 | Search modes - Find all my search terms | 59,743 |  |
| S19 | (MM "Stress, Psychological") | Search modes - Find all my search terms | 31,847 |  |
| S20 | (MM "Stress, Physiological") | Search modes - Find all my search terms | 2,946 |  |
| S21 | (MM "Biological Markers") | Search modes - Find all my search terms | 19,537 |  |
| S22 | TI ( (allostatic N2 (load* or overload* or mediator*)) ) OR AB ( (allostatic N2 (load* or overload* or mediator*)) ) | Search modes - Find all my search terms | 567 |  |
| S23 | TI allostasis OR AB allostasis | Search modes - Find all my search terms | 90 |  |
| S24 | TI ( (stress N3 (chronic or cumulative or antenatal or ante-natal or prenatal or pre-natal or psychosocial or maternal or psychologic* or physiologic*)) ) OR AB ( (stress N3 (chronic or cumulative or antenatal or ante-natal or prenatal or pre-natal or psychosocial or maternal or psychologic* or physiologic*)) ) | Search modes - Find all my search terms | 13,991 |  |
| S25 | TI ( (cumulative N3 ("physiologic* risk" or "biologic* risk" or "psychologic* risk")) ) OR AB ( (cumulative N3 ("physiologic* risk" or "biologic* risk" or "psychologic* risk")) ) | Search modes - Find all my search terms | 24 |  |
| S26 | TI ( (biomarker* N6 (maternal or pregnan* or perinatal or prenatal or pre-natal or peri-natal or pre-natal or pre-conception or preconception or stress)) ) OR AB ( (biomarker* N6 (maternal or pregnan* or perinatal or prenatal or pre-natal or peri-natal or pre-natal or pre-conception or preconception or stress)) ) | Search modes - Find all my search terms | 2,771 |  |
| S27 | TI ( (“biological marker*” N6 (maternal or pregnan* or perinatal or prenatal or pre-natal or peri-natal or pre-natal or pre-conception or preconception or stress)) ) OR AB ( (“biological marker*” N6 (maternal or pregnan* or perinatal or prenatal or pre-natal or peri-natal or pre-natal or pre-conception or preconception or stress)) ) | Search modes - Find all my search terms | 86 |  |
| S28 | TI ( (two-hit* or “multiple hit*”) ) OR AB ( (two-hit* or “multiple hit*”) ) | Search modes - Find all my search terms | 270 |  |
| S29 | S19 OR S20 OR S21 OR S22 OR S23 OR S24 OR S25 OR S26 OR S27 OR S28 | Search modes - Find all my search terms | 65,390 |  |
| S30 | S18 AND S29 | Search modes - Find all my search terms | 1,386 | |
| S31 | S18 AND S29 | Narrow by Language: - english  Search modes - Find all my search terms | 1,347 | |

**Scopus (Elsevier)**

( ( ( ( TITLE-ABS-KEY ( ( *birth*  OR  *births*  OR  *childbirth*  OR  *born*  OR  *delivery*  OR  *delivered*  OR  *deliveries*  OR  *parturition*  OR  *labor*  OR  *labour* ) )  OR  TITLE-ABS-KEY ( ( *infant**  OR  *baby*  OR  *babies*  OR  *newborn**  OR  *neonate** ) ) ) )  AND  ( ( TITLE-ABS-KEY ( ( ( *"24"*  OR  *"25"*  OR  *"26"*  OR  *"27"*  OR  *"28"*  OR  *"29"*  OR  *"30"*  OR  *"31"*  OR  *"32"*  OR  *"33"*  OR  *"34"*  OR  *"35"*  OR  *"36"* )  W/1  ( *week**  OR  *wk**  OR  *ga*  OR  *gestation**  OR  *"postmenstrual age"*  OR  *"post-menstrual age"* ) ) )  OR  TITLE-ABS-KEY ( ( *24wk**  OR  *25wk**  OR  *26wk**  OR  *27wk**  OR  *28wk**  OR  *29wk**  OR  *30wk**  OR  *31wk**  OR  *32wk**  OR  *33wk**  OR  *34wk**  OR  *35wk**  OR  *36wk** ) )  OR  TITLE-ABS-KEY ( ( *24week**  OR  *25week**  OR  *26week**  OR  *27week**  OR  *28week**  OR  *29week**  OR  *30week**  OR  *31week**  OR  *32week**  OR  *33week**  OR  *34week**  OR  *35week**  OR  *36week** ) )  OR  TITLE-ABS-KEY ( ( ( *"less than"*  OR  *before*  OR  *"fewer than"* )  W/5  ( *"37 week*"*  OR  *"37week*"*  OR  *"37wk*"*  OR  *"37 wk*"* ) ) )  OR  TITLE-ABS-KEY ( ( *"36 6/7 week*"*  OR  *"36 6/7 wk*"*  OR  *"36 6/7week*"*  OR  *"36 6/7wk*"* ) ) ) ) )  OR  ( ( TITLE-ABS-KEY ( ( ( *preterm**  OR  *pre-term**  OR  *prematur**  OR  *pre-matur** )  W/5  ( *birth*  OR  *births*  OR  *childbirth*  OR  *born*  OR  *delivery*  OR  *delivered*  OR  *deliveries*  OR  *parturition*  OR  *labor*  OR  *labour* ) ) )  OR  TITLE-ABS-KEY ( ( ( *early-preterm*  OR  *mild-preterm*  OR  *near-term* )  W/5  ( *birth*  OR  *births*  OR  *childbirth*  OR  *born*  OR  *delivery*  OR  *delivered*  OR  *deliveries*  OR  *parturition*  OR  *labor*  OR  *labour* ) ) ) ) ) )  AND  ( ( ( TITLE-ABS-KEY ( ( *allostatic*  W/2  ( *load**  OR  *overload**  OR  *mediator** ) ) )  OR  TITLE-ABS-KEY ( *allostasis* ) ) )  OR  ( TITLE-ABS-KEY ( ( *stress*  W/3  ( *chronic*  OR  *cumulative*  OR  *antenatal*  OR  *ante-natal*  OR  *prenatal*  OR  *pre-natal*  OR  *psychosocial*  OR  *maternal*  OR  *psychologic**  OR  *physiologic** ) ) ) )  OR  ( TITLE-ABS-KEY ( ( *cumulative*  W/3  ( *"physiologic* risk"*  OR  *"biologic* risk"*  OR  *"psychologic* risk"* ) ) ) )  OR  ( TITLE-ABS-KEY ( ( *biomarker**  W/6  ( *maternal*  OR  *pregnan**  OR  *perinatal*  OR  *prenatal*  OR  *pre-natal*  OR  *peri-natal*  OR  *pre-natal*  OR  *pre-conception*  OR  *preconception*  OR  *stress* ) ) ) )  OR  ( TITLE-ABS-KEY ( ( *"biological marker*"*  W/6  ( *maternal*  OR  *pregnan**  OR  *perinatal*  OR  *prenatal*  OR  *pre-natal*  OR  *peri-natal*  OR  *pre-natal*  OR  *pre-conception*  OR  *preconception*  OR  *stress* ) ) ) )  OR  ( TITLE-ABS-KEY ( ( *two-hit**  OR  *"multiple hit*"* ) ) ) )  AND  ( LIMIT-TO ( LANGUAGE ,  *"English"* ) )  AND  ( EXCLUDE ( DOCTYPE ,  *"ch"* )  OR  EXCLUDE ( DOCTYPE ,  *"le"* )  OR  EXCLUDE ( DOCTYPE ,  *"ed"* ) )

**Web of Science** Including:

Science Citation Index Expanded 1900 – present

Social Sciences Citation Index 1900 – present

Arts & Humanities Citation Index 1975 – present

Conference Proceedings Citation Index – Science 1990 – present

Conference Proceedings Citation Index – Social Sciences 1990 – present

Emerging Sources Citation Index – 2017 - present

| 14 | #13 limited to English | 2,209 |
| --- | --- | --- |
| 13 | **#11 AND #12** | 2,252 |
| 12 | **#4 OR #5 OR #6 OR #7 OR #8 OR #9** | 116,848 |
| 11 | **#10 OR #1** | 113,227 |
| 10 | **#2 AND #3** | 57,041 |
| 9 | **(two-hit* or multiple hit*)** (Topic) | 12,688 |
| 8 | **(biological marker* NEAR/6 (maternal or pregnan* or perinatal or prenatal or pre-natal or peri-natal or pre-natal or pre-conception or preconception or stress))** (Topic) | 4,332 |
| 7 | **biomarker* NEAR/6 (maternal or pregnan* or perinatal or prenatal or pre-natal or peri-natal or pre-natal or pre-conception or preconception or stress)** (Topic) | 15,665 |
| 6 | **cumulative NEAR/3 (physiologic* or biologic* or psychologic*) NEAR/3 risk*** (Topic) | 216 |
| 5 | stress NEAR/3 (chronic or cumulative or antenatal or ante-natal or prenatal or pre-natal or psychosocial or maternal or psychologic* or physiologic*) (Topic) | 83,528 |
| 4 | **(allostatic NEAR/2 (load* or overload* or mediator*))** (Topic) or **Allostasis** (Topic) | 3,798 |
| 3 | (birth or births or childbirth or born or delivery or delivered or deliveries or parturition or labor or labour) (Topic) or (infant* or baby or babies or newborn* or neonate*) (Topic) | 3,057,760 |
| 2 | ("24" or "25" or "26" or "27" or "28" or "29" or "30" or "31" or "32" or "33" or "34" or "35" or "36") NEAR/1 (week* or wk or wks or ga or gestation* or "postmenstrual age" or "post-menstrual age") (Topic) or (24wk* or 25wk* or 26wk* or 27wk* or 28wk* or 29wk* or 30wk* or 31wk* or 32wk* or 33wk* or 34wk* or 35wk* or 36wk*) (Topic) or (24week* or 25week* or 26week* or 27week* or 28week* or 29week* or 30week* or 31week* or 32week* or 33week* or 34week* or 35week* or 36week*) (Topic) or ("less than" or before or "fewer than") NEAR/5 ("37 week*" or "37week*" or "37wk*" or "37 wk" or "37 wks") (Topic) or ("36 6/7 week*" or "36 6/7wk*" or "36 6/7week*" or "36 6/7wk*") (Topic) | 139,919 |
| 1 | ((preterm* or pre-term* or prematur* or pre-matur*) NEAR/5 (birth or births or childbirth or born or delivery or delivered or deliveries or parturition or labor or labour)) (Topic) or ((early-preterm or mild-preterm or near-term) NEAR/5 (birth or births or childbirth or born or delivery or delivered or deliveries or parturition or labor or labour)) (Topic) | 73,974 |
