# Supplementary material for: Prenatal allostatic load and preterm birth: A systematic review
Source: Front Psychol. 2022 Oct 4;13:1004073. doi: 10.3389/fpsyg.2022.1004073 (PMC9577361; doi:10.3389/fpsyg.2022.1004073)
Supplement: Supplementary file 3 [file Data_Sheet_3.docx]

Supplementary Material S3: Study risk of bias assessment using the QUIPS tool (Hayden et al., 2013)

|  | McKee et al. (2017) | Sayre (2016) | Wallace and Harville (2013) |
| --- | --- | --- | --- |
| Study participation | Moderate to high | High | High |
| Study attrition | Low | High | Moderate to high |
| Prognostic factor measurement | High | High | Low |
| Outcome measurement | Low to moderate | Moderate to high | Low |
| Study confounding | Low | Moderate | Moderate |
| Statistical analysis and reporting | Moderate to high | High | Moderate |
