# Supplementary material for: Prenatal allostatic load and preterm birth: A systematic review
Source: Front Psychol. 2022 Oct 4;13:1004073. doi: 10.3389/fpsyg.2022.1004073 (PMC9577361; doi:10.3389/fpsyg.2022.1004073)
Supplement: Supplementary file 4 [file Data_Sheet_4.docx]

Supplementary Material S4: Stated inclusion and exclusion criteria at enrolment in included studies

|  | Inclusion criteria | Exclusion criteria |
| --- | --- | --- |
| McKee et al. (2017) | - Women aged 18–35 years - <28 weeks’ gestation - Planned delivery at one of four hospitals in Monroe County, New York, USA - Singleton pregnancy - English literacy | - BMI >35 kg/m^2^ - Blood pressure treated with drug therapy - Other chronic diseases - Miscarriage and stillbirth |
| Sayre (2016) | - Women aged 16 years or older - Singleton pregnancy | - History of diabetes or heart disease - “Indication of drug abuse” (p. 29) in second or third trimesters - Diagnosis of STBBI in second trimester - Multifetal pregnancy - For multigravid women, history of pregnancies with complications or preterm birth |
| Wallace and Harville (2013) | - Women aged 20–35 years - Singleton pregnancy - Self-identified as white or African-American - English-speaking - Receiving care with planned delivery at Tulane-Lakeside Hospital in Metairie, Louisiana, USA | NA |

BMI, body mass index; STBBI, sexually transmitted and bloodborne infection; USA, United States of America.
